# Supplementary material for: Acetylation-induced TDP-43 pathology is suppressed by an HSF1-dependent chaperone program
Source: Nat Commun. 2017 Jul 19;8:82. doi: 10.1038/s41467-017-00088-4 (PMC5517419; doi:10.1038/s41467-017-00088-4)
Supplement: Supplementary file 1 — Supplementary Information [file 41467_2017_88_MOESM1_ESM.pdf]

File name: Supplementary Information
Description: Supplementary figures and supplementary table.

File name: Peer review file
Description:

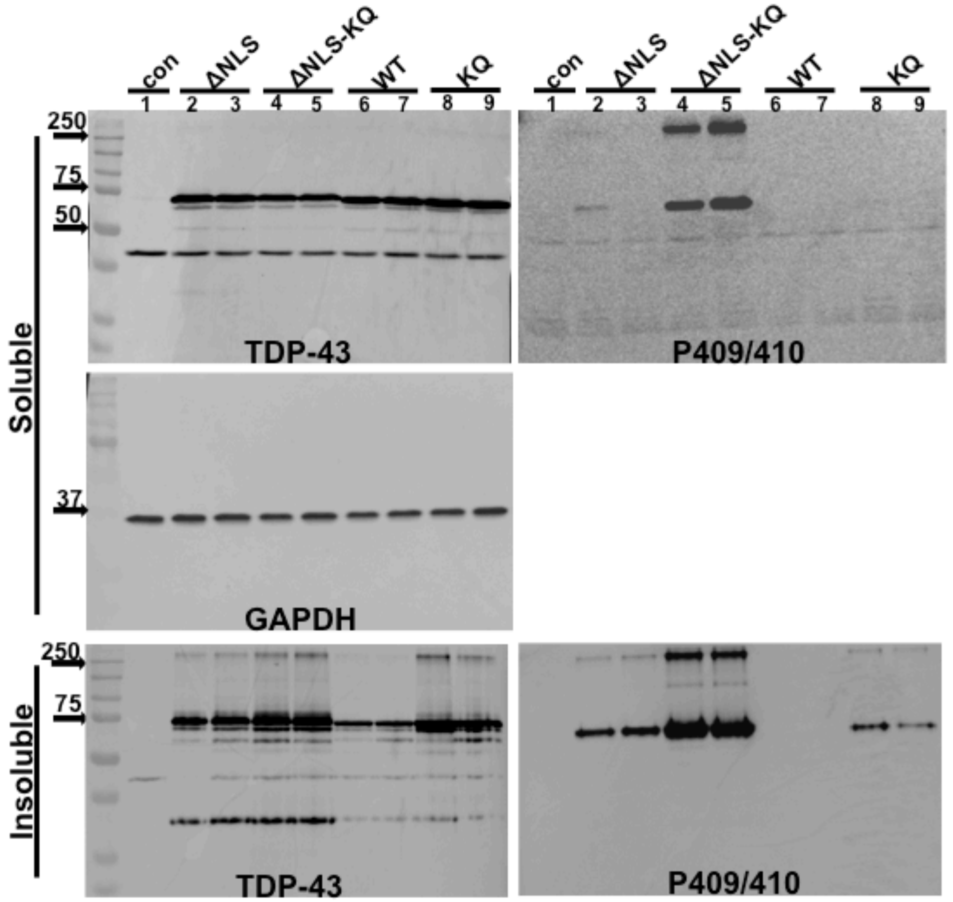

Supplementary Figure 1: Full-size immunoblots used to generate the cropped images shown in Fig. 1

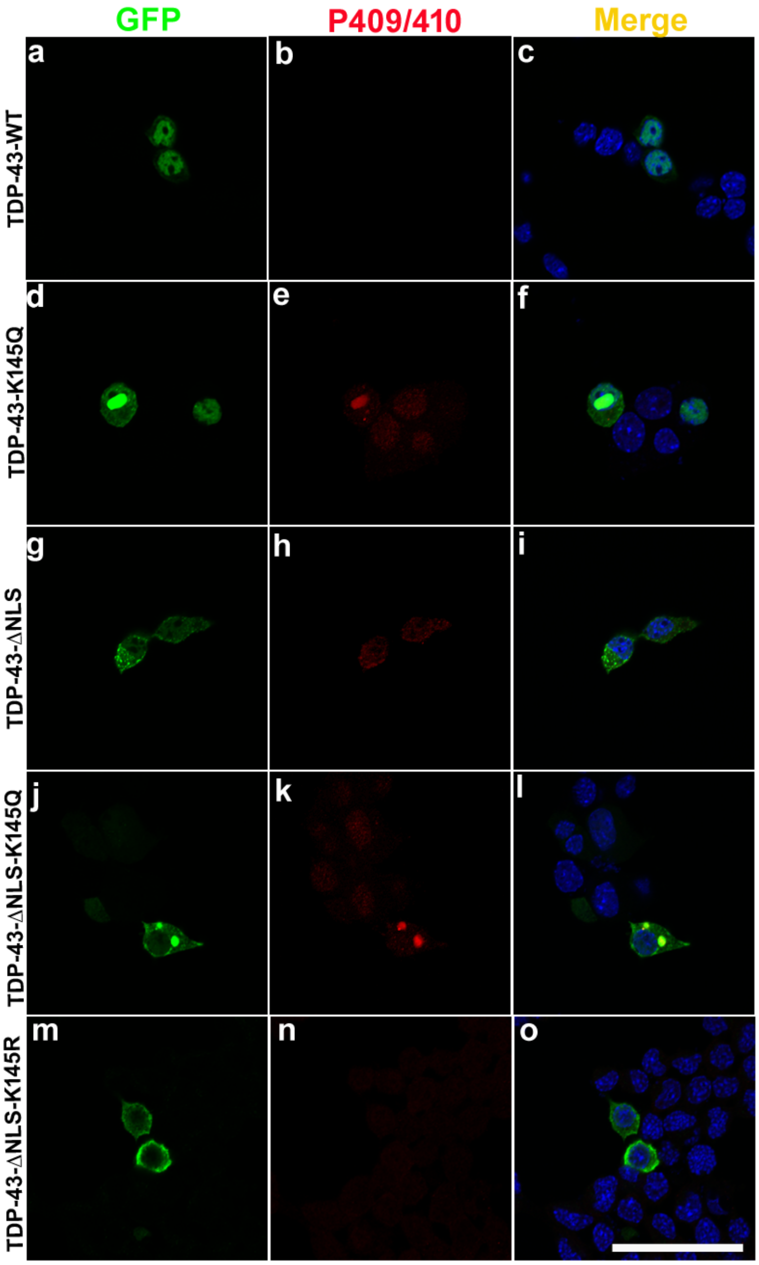

Supplementary Figure 2: Expression of a nuclear or cytoplasmic targeted TDP-43 K145Q mutation promotes TDP-43 inclusion formation in motor neuron-like NSC-34 cells

TDP-43 plasmids expressing TDP-43-WT (**a-c**), TDP-43-K145Q (**d-f**), TDP-43--ΔNLS (**g-i**), TDP-43-ΔNLS-K145Q (**j-l**), or TDP-43-ΔNLS-K145R (**m-o**) were transfected into NSC-34 cells and 48 hr later cells were analyzed by confocal microscopy using GFP and P409/410 phospho-TDP-43 antibodies. DAPI was used to mark nuclei. Only cells expressing TDP-43-K145Q or TDP-43-ΔNLS-K145Q mutants showed perinuclear aggregates that were hyper-phosphorylated. K→R non-mimic mutants were not aggregate-prone. Scale bar = 50 μm.

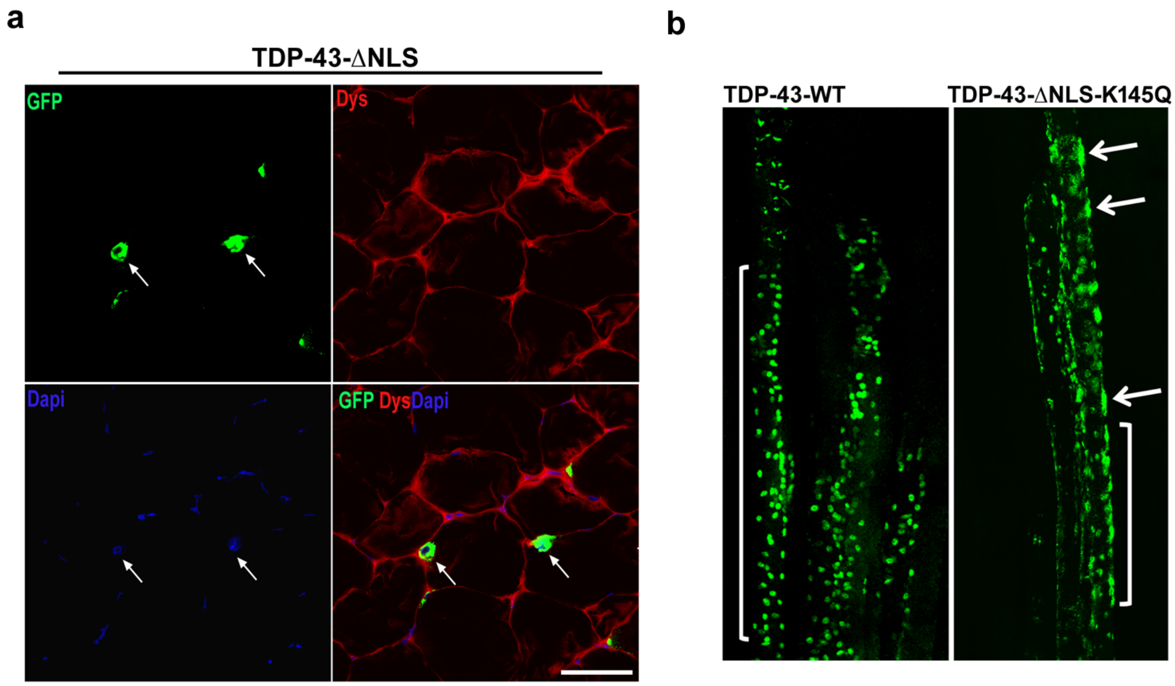

Supplementary Figure 3: Cytoplasmic targeted TDP-43 shows increased aggregation, with TDP-43-ΔNLS accumulating around nuclei and TDP-43-ΔNLS-K145Q accumulating as aggregated deposits at the myofiber periphery/membrane

a) Muscle sections from mouse tibialis anterior (TA) muscles electroporated with GFP-tagged TDP-43-ΔNLS plasmid for 4 days were double-labeled with GFP (green) and Dystrophin (Dys, red) antibodies. Nuclei were stained with DAPI (blue). TDP-43-ΔNLS (green) accumulated in the cytoplasm and immediately surrounding, or adjacent to, the nuclear membrane (white arrows). This pattern was not observed with TDP-43-WT (see Fig. 2). Scale bar = 50 μm. **b)** Longitudinal muscle sections from TDP-43-WT or TDP-43-ΔNLS-K145Q expressing muscles were visualized with GFP antibodies. While TDP-43-WT localized exclusively to nuclei as expected (see nuclei within the white bracket, left panel), expression of TDP-43-ΔNLS-K145Q led to aggregate accumulation that was prominent along the edge of the muscle membrane (see white arrows and white bracket, right panel). Images were acquired at 20X magnification on a LSM780 confocal laser microscope (Carl Zeiss).

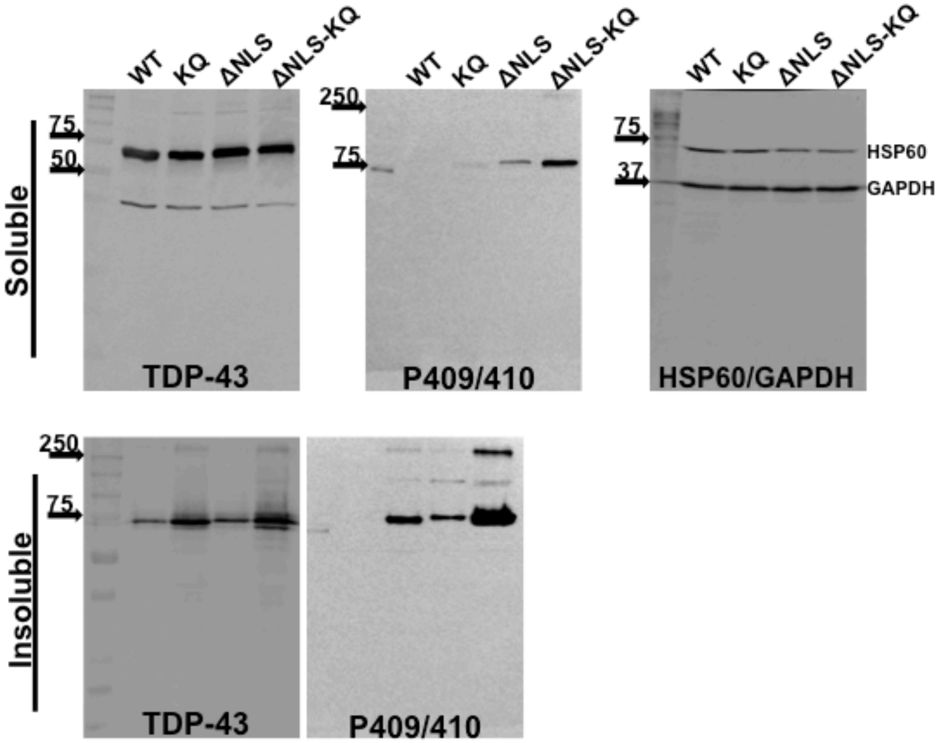

Supplementary Figure 4: Full-size immunoblots used to generate the cropped images shown in Fig. 2

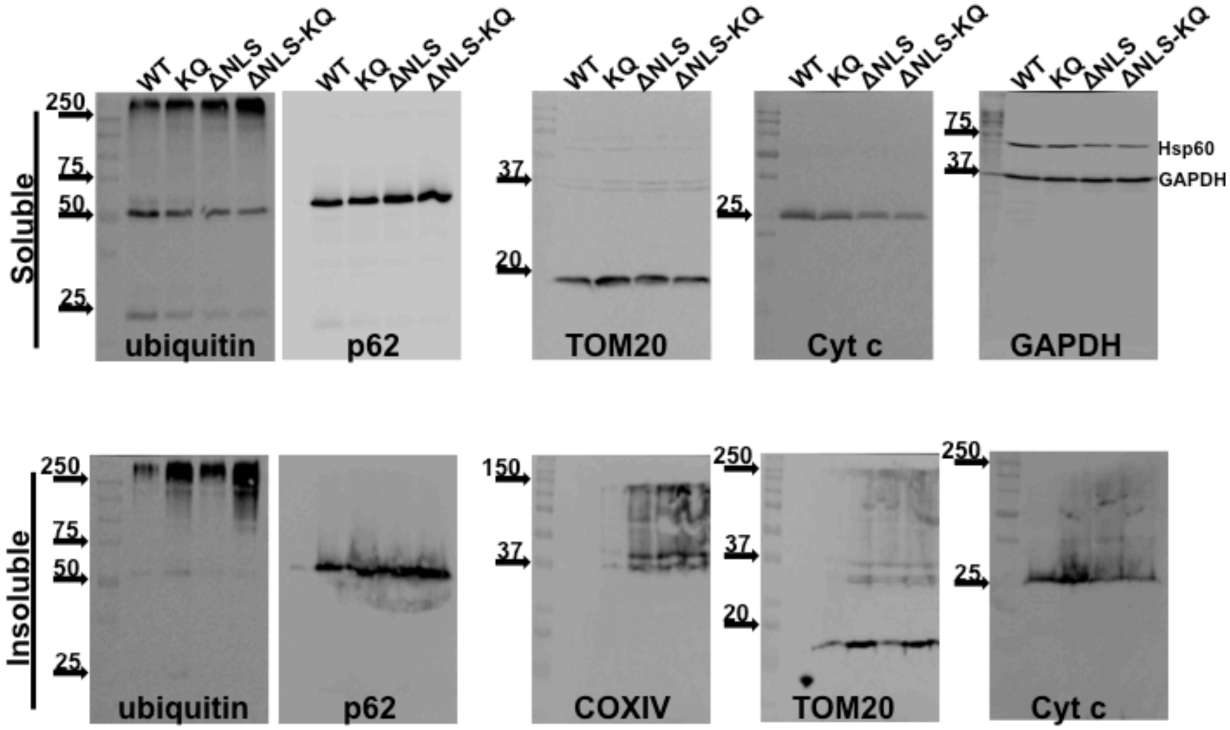

Supplementary Figure 5: Full-size immunoblots used to generate the cropped images shown in Fig. 3

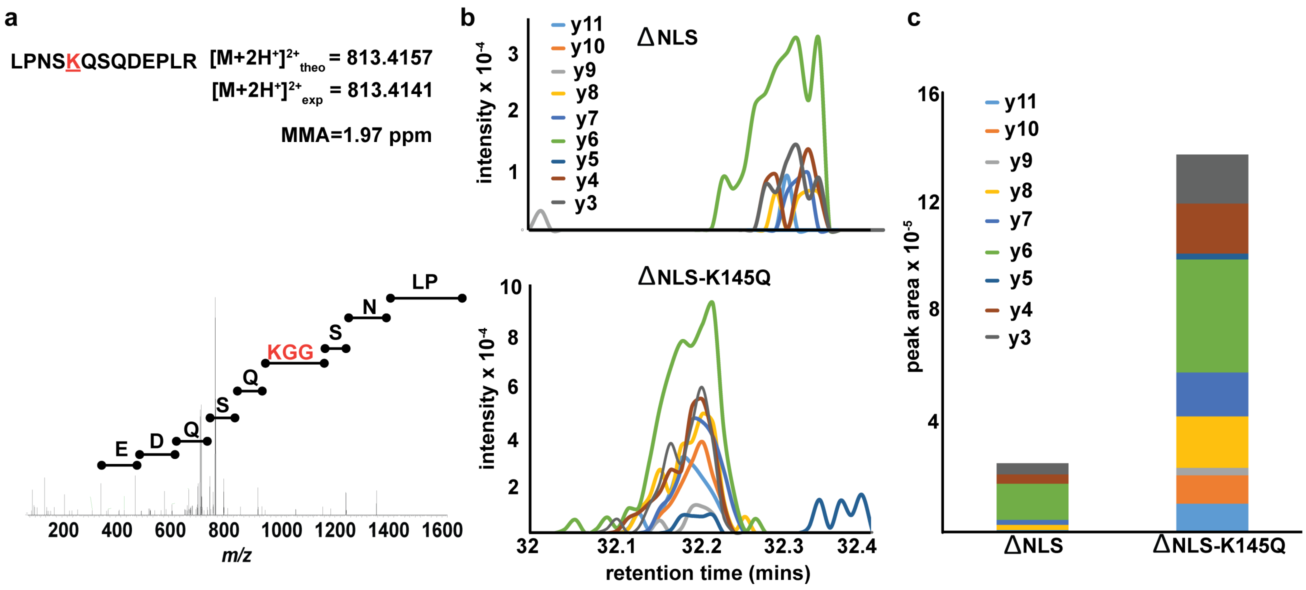

Supplementary Figure 6: Mass spectrometry analysis identified ubiquitinated residue K181 in the TDP-43 RRM1 domain

QBI-293 cells were transiently transfected with cytoplasmic-localized TDP-43-ΔNLS or TDP-43-ΔNLS-K145Q mutant. Total lysates were prepared, as described in the methods, and TDP-43 was immunoprecipitated using anti-TDP-43 (clone 171) that was complexed to protein A/G beads (Sigma). Samples were digested after elution using the filter aided sample preparation method and subjected to both discovery and targeted proteomics via LC MS/MS on a quadrupole orbitrap. **a)** A spectrum of the TDP-43 peptide, with the y ion series annotated. ^{177}LPNS**K**QSQDEPLR^{189} was identified containing ubiquitinated residue K181 within the TDP-43 RRM1 region. **b)** PRM analysis of the peptide provides further confirmation of identification. **c)** Integrated peptide intensities of the PRM data between TDP-43-ΔNLS and TDP-43-ΔNLS K145Q samples.

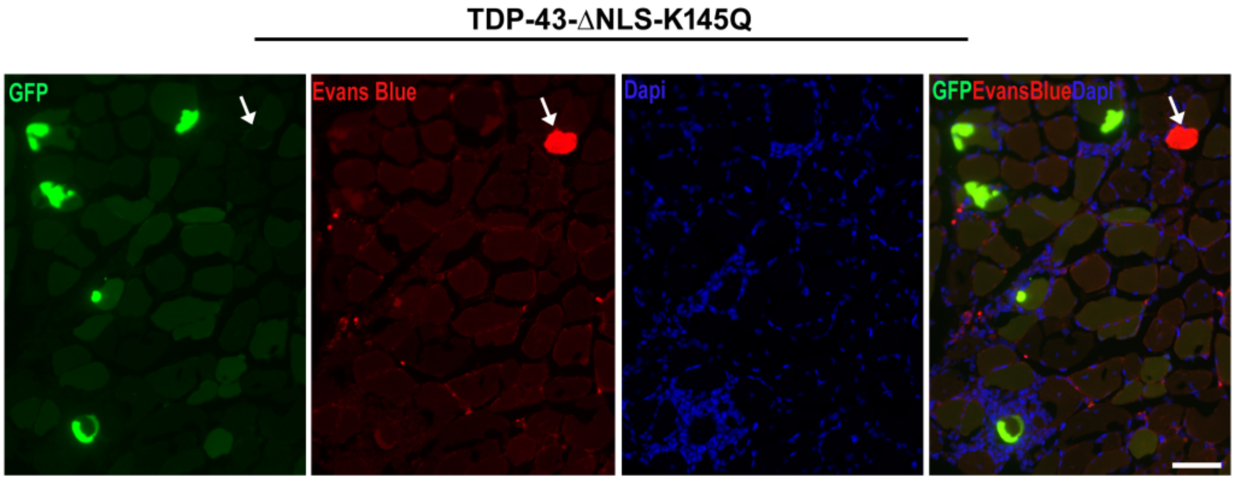

Supplementary Figure 7: Rare EBD-positive fibers were identified in electroporated or non-electroporated myofibers, irrespective of ectopic expression of TDP-43-ΔNLS-K145Q

Evans Blue dye (EBD) was intraperitoneal (i.p.) injected for 24 hr into mice that had been electroporated with TDP-43-ΔNLS-K145Q for 21 days prior to muscle tissue harvest (see methods). Cross-sections from these muscles were analyzed for GFP (green) and EBD fluorescence (red, 594 nm wavelength). Nuclei were counterstained with DAPI (blue). EBD-positive myofibers were very rare (white arrows) and did not co-localize with large TDP-43 inclusions (see merge). Scale bar = 50 µm.

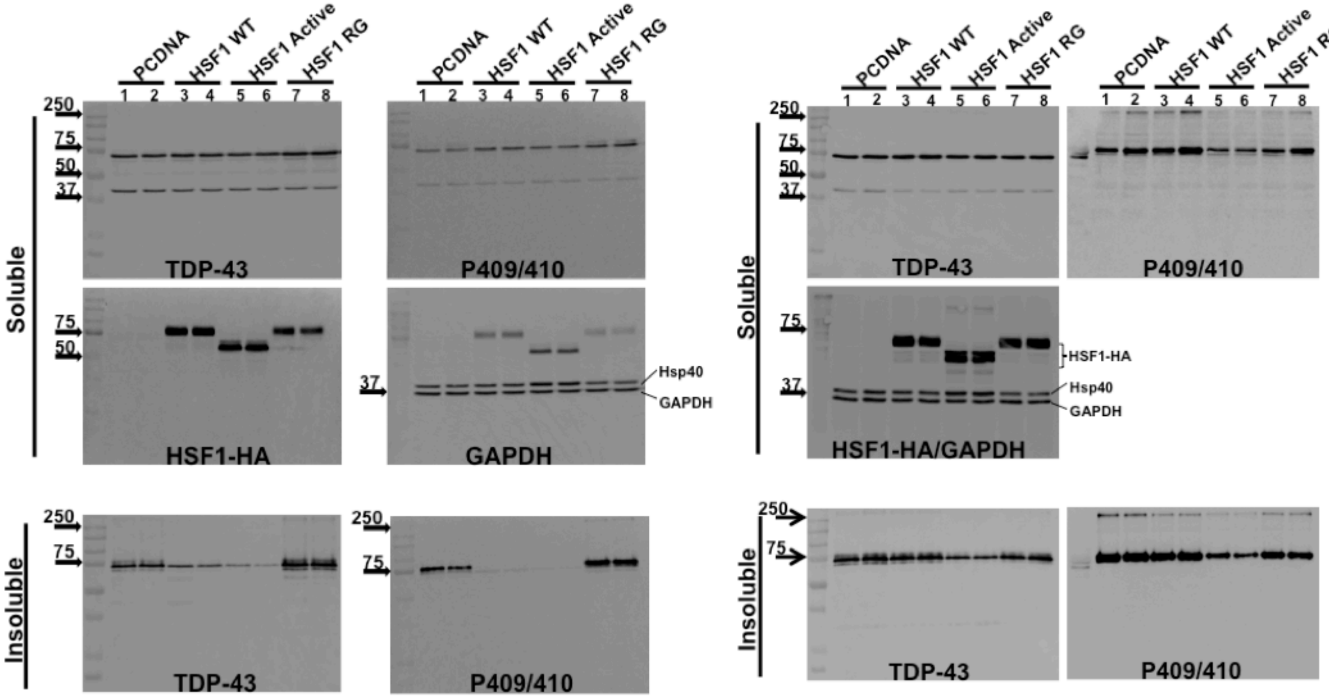

Supplementary Figure 8: Full-size immunoblots used to generate the cropped images shown in Fig. 6. Left panel, from Figs 6b and 6c; right panel, from Figs 6f and 6g.

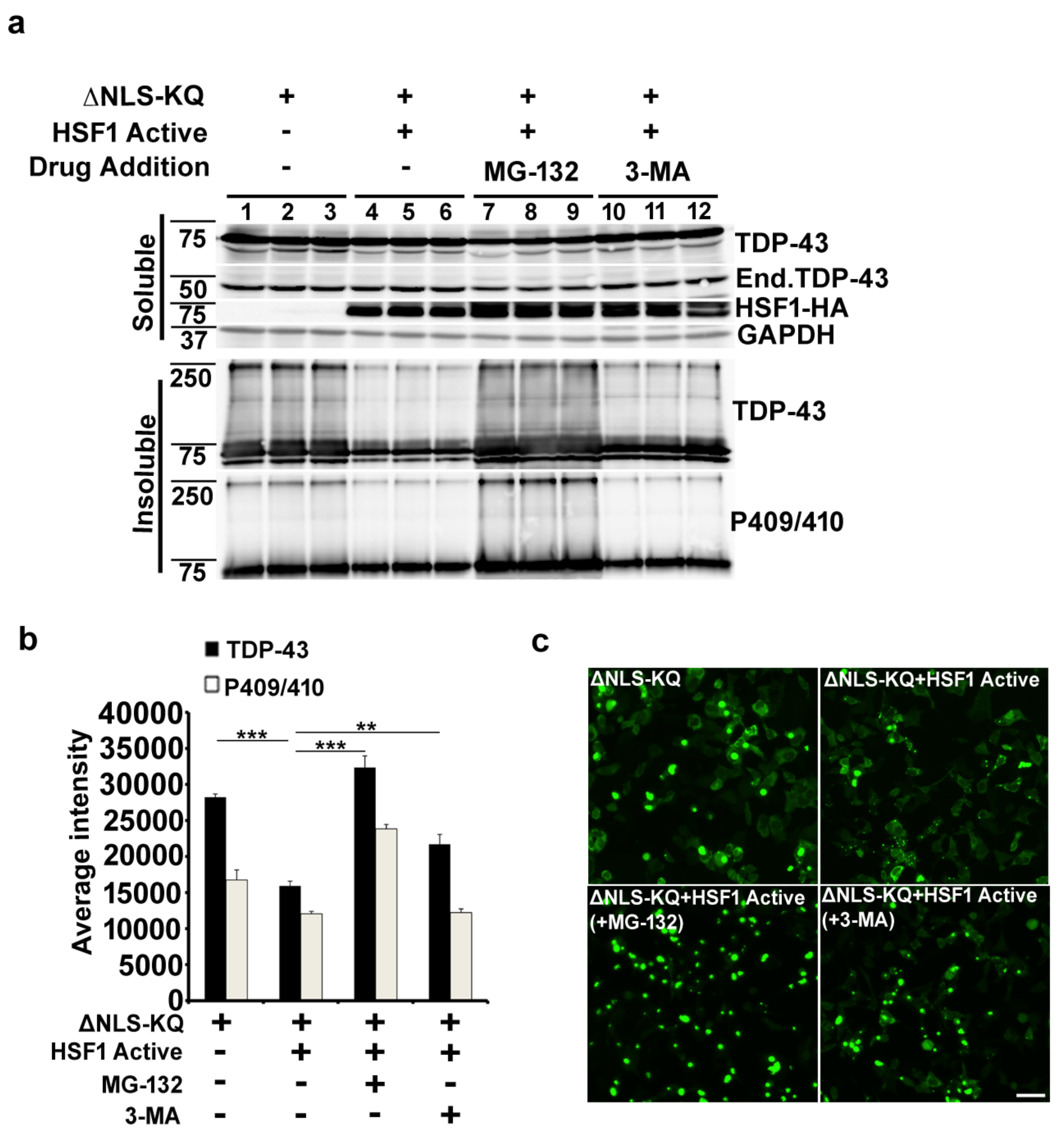

Supplementary Figure 9: HSF1-mediated disaggregation of TDP-43 leads to its degradation, in part, via proteasome and autophagy-dependent mechanisms

a) QBI-293 cells transfected with TDP-43-ΔNLS-K145Q (ΔNLS-KQ) were then sequentially transfected with an active HSF1 construct (transfected ~12 hr later, introduced after the initial TDP-43 transfection, as described in the methods). Cells were treated with either a proteasome inhibitor (MG-132, 1 μM) or an autophagy inhibitor (3-MA, 10 mM) to block TDP-43 degradation. Homogenates were analyzed by immunoblotting using the indicated antibodies. **b)** Average band intensities were quantified to evaluate the accumulation of insoluble TDP-43 in response to drug treatments. **c)** Prior to harvest, cells were analyzed by fluorescence microscopy to visualize aggregates, which were induced by MG-132 or 3-MA treatment. Error bars indicate s.e.m, and the asterisk indicates statistical significance with p-value < 0.001(***) and p-value < 0.01(**) as measured by student t-test from N=3 biological replicates. Scale bar = 50 μm.

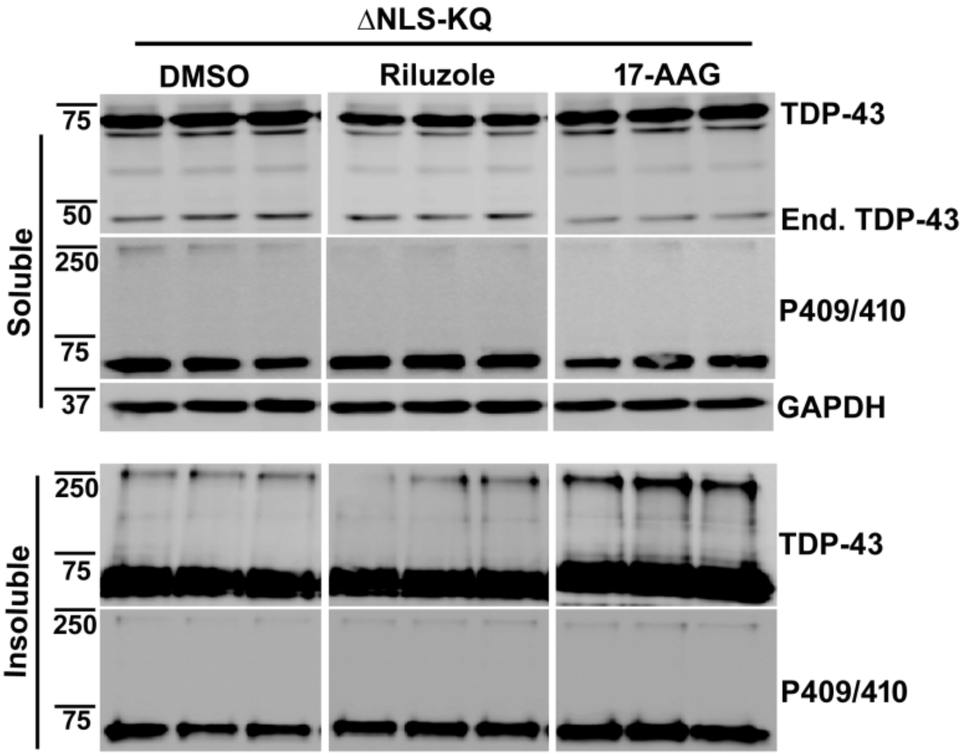

Supplementary Figure 10: **The Hsp90 inhibitor 17-AAG and FDA-approved ALS drug riluzole showed no apparent suppression of insoluble TDP-43-ΔNLS-K145Q aggregates**

QBI-293 cells expressing the aggregate-prone TDP-43-ΔNLS-K145Q (ΔNLS-KQ) were exposed to DMSO (vehicle control), riluzole, or 17-AAG (5 μM each) for 24 hr. Soluble and insoluble fractions isolated from these cells were analyzed by immunoblotting using TDP-43, phospho-TDP-43 (P409/410) or GAPDH antibodies. Shown is a representative image in which reduction of TDP-43 aggregates was not observed in response to these drugs. In contrast, HSF1A significantly reduced the insoluble accumulation of TDP-43 (see Fig. 6).

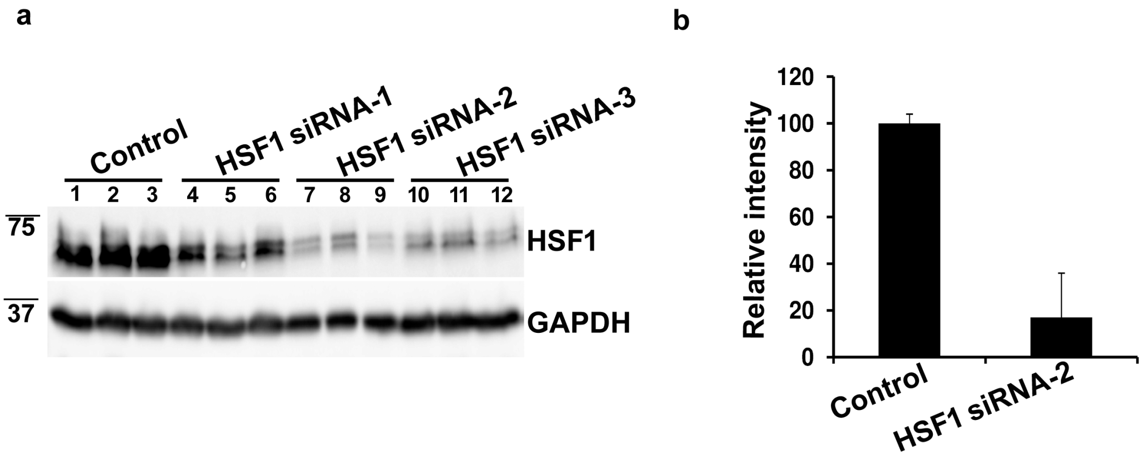

Supplementary Figure 11: HSF1 protein levels were reduced by HSF-1 siRNA-mediated knock-down

a) Neuro2A cells were transfected with control siRNA or three independent HSF1-specific siRNA duplexes targeting mouse HSF1 (ThermoFisher, HSF1 siRNAs #1-3) for 3 days and analyzed by immunoblotting using a rat monoclonal HSF1antibody (Enzo Life Sciences). **b)** Relative band intensities were quantified to evaluate siRNA-mediated knock-down of HSF1 relative to GAPDH levels. HSF1 siRNA#2 reduced HSF1 levels by ~90% compared to control and was chosen for subsequent electroporation experiments *in vivo* (Fig. 7). Error bars indicate s.e.m.

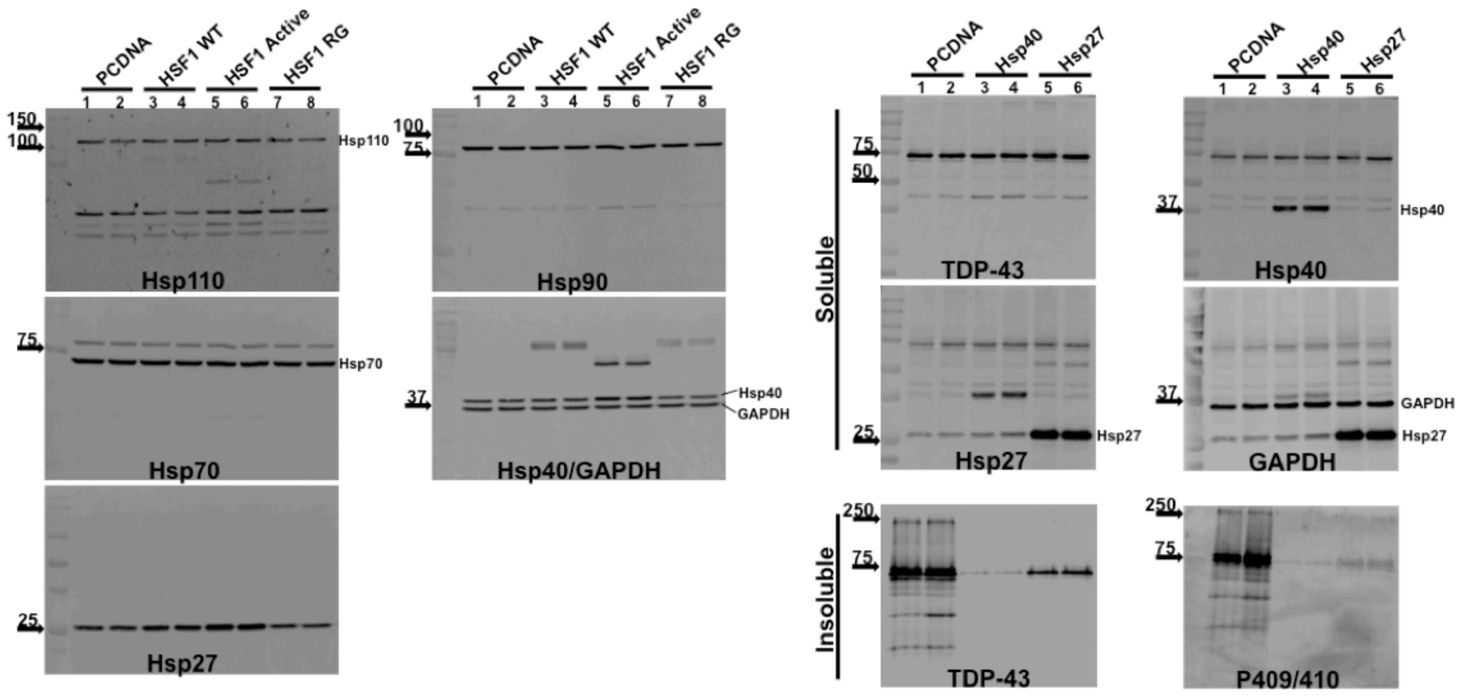

Supplementary Figure 12: Full-size immunoblots used to generate the cropped images shown in Fig. 8. Left panel, from Figs 8a and 8b; right panel, from Fig. 8e.

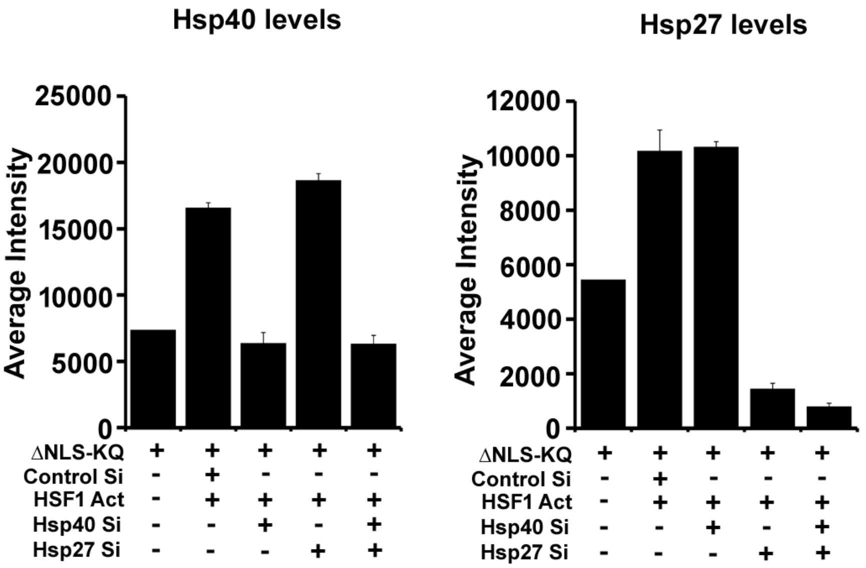

Supplementary Figure 13: Hsp40 and Hsp27 protein levels were reduced by siRNA-mediated knock-down in the presence or absence of TDP-43 and HSF1 constructs

Cells co-transfected with TDP-43-ΔNLS-K145Q and active HSF1 were treated with either a scrambled control siRNA, Hsp40 siRNA, Hsp27 siRNA, or a combined (Hsp40+27) siRNA mixture, as described in the methods. Cell lysates were analyzed by immunoblotting using Hsp40 and Hsp27 antibodies. Average band intensities were quantified to evaluate the extent of siRNA-mediated knock-down of Hsp40 and Hsp27. The siRNA validation described here was required for assessment of HSF1-mediated disaggregation described in Figs 9a and 9b. Error bars indicate s.e.m.

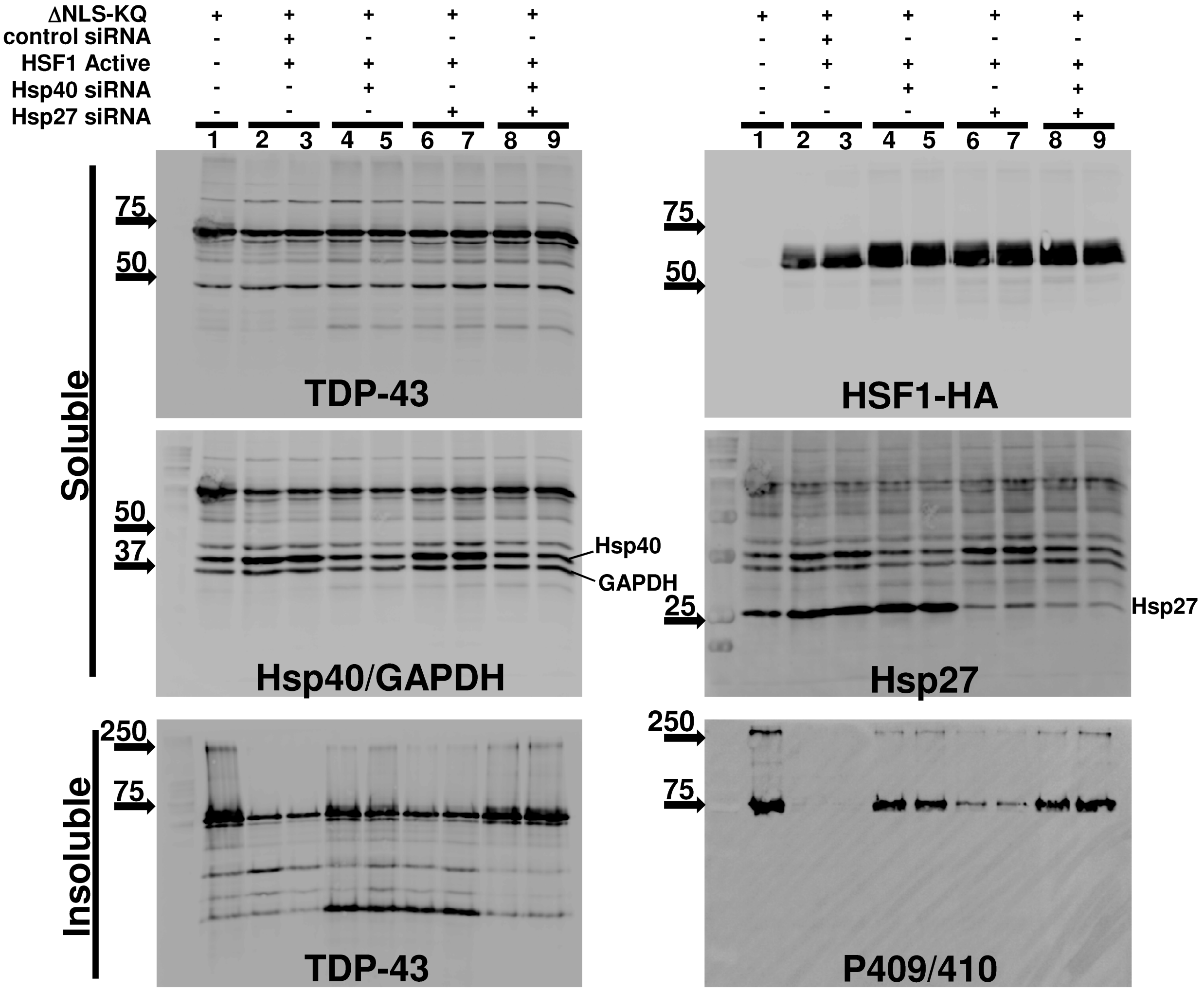

Supplementary Figure 14: Full-size immunoblots used to generate the cropped images shown in Fig. 9.

A. Primers used for mutagenesis			
**Gene**	**Mutation**	**Forward primer**	**Reverse primer**
HSF-1	R71G	CATGGCCAGCTTCGTGGGGCAGCTCAACATGTATG	CATACATGTTGAGCTGCCCCACGAAGCTGGCCATG
HSF-1	deletion of residues 221-315	GGGCGCCCATCTTCCGTG	ATGTGCTGAGCCACTGTCG
HSF-1	L395E	GCTATGGACTCCAACGAGGATAACCTGCAGACC	GGTCTGCAGGTTATCCTCGTTGGAGTCCATAGC
TDP-43	K145Q	AAGACTGGTCATTCACAGGGGTTTGGCTTTGTTC	GAACAAAGCCAAACCCCTGTGAATGACCAGTCTT
TDP-43	K145R	CTTAAGACTGGTCATTCAAGGGGGTTTGGCTTTGTTCG	CGAACAAAGCCAAACCCCCTTGAATGACCAGTCTTAAG
**B. siRNA used for gene expression knockdown**			
**Gene targeted**	**Catalogue No.**	**Description/Sequence**	
HSP40 (h)	sc-35599 (Santa Cruz)	a pool of 3 target-specific 19-25 nt	
HSP27 (h)	sc-29350 (Santa Cruz)	a pool of 3 target-specific 19-25 nt	
HSF-1 (#1) (m)	10620318-285251 E09 (Thermofisher)	GGAAACAGGAGUGUAUGGACUCCAA	
HSF-1 (#2) (m)	10620318-285251 E011 (Thermofisher)	GCUCUGGACCCAUAAUCUCCGAUAU	
HSF-1 (#3) (m)	10620318-285251 E07 (Thermofisher)	ACACCGAGUUCCAGCAUCCUUGUUU	

Supplementary Table 1: Description of mutagenesis and siRNA primer sequences used in this study
